# Possible Correlations between Atherosclerosis, Acute Coronary Syndromes and COVID-19

**DOI:** 10.3390/jcm9113746

**Published:** 2020-11-21

**Authors:** Oliwia Grzegorowska, Jacek Lorkowski

**Affiliations:** 1 Department of Cardiology, Independent Public Regional Hospital, 71-455 Szczecin, Poland; 2 Department of Orthopaedic and Traumatology, Central Clinical Hospital of Ministry of Interior, 02-507 Warsaw, Poland; jacek.lorkowski@gmail.com; 3 Faculty of Health Sciences, Medical University of Mazovia, 01-793 Warsaw, Poland

**Keywords:** COVID-19, SARS-CoV-2, atherosclerosis, cardiovascular, acute coronary syndrome, inflammation

## Abstract

An outbreak of SARS-CoV-2 infection in December 2019 became a major global concern in 2020. Since then, several articles analyzing the course, complications and mechanisms of the infection have appeared. However, there are very few papers explaining the possible correlations between COVID-19, atherosclerosis and acute coronary syndromes. We performed an analysis of PubMed, Cochrane, Google Scholar, and MEDLINE databases. As of September 15, 2020, the results were as follows: for “COVID-19” and “cardiovascular system” we obtained 687 results; for “COVID-19” and “myocardial infarction” together with “COVID-19” and “acute coronary syndrome” we obtained 328 results; for “COVID-19” and “atherosclerosis” we obtained 57 results. Some of them did not fulfill the search criteria or concerned the field of neurology. Only articles written in English, German and Polish were analyzed for a total number of 432 papers. While the link between inflammatory response, COVID- 19 and atherosclerosis still remains unclear, there is evidence that suggests a more likely correlation between them. Practitioners’ efforts should be focused on the prevention of excessive inflammatory response and possible complications, while there are limited specific therapeutic options against SARS-CoV-2. Furthermore, special attention should be paid to cardioprotection during the pandemic.

## 1. Introduction

An outbreak of SARS-CoV-2 infection first identified in Wuhan, China, in December 2019 became a major global concern in 2020. Since then, several articles analyzing the course, complications and mechanisms of the infection have appeared. Multiple studies describing involvement of the cardiovascular system and management of cardiovascular complications are available. However, there are only a few papers explaining possible correlations between COVID-19, atherosclerosis and acute coronary syndromes. The topic is of utmost importance given the possibility of reducing the COVID-19 mortality rate. In this case, creating evidence-based algorithms improves effectiveness and therefore speeds up the thought process and results in increased survival rates.

There are four groups of coronaviruses: alpha, beta, gamma and delta. Until now, six types, all of which belong to alpha or beta groups, were known. SARS-CoV-2, like SARS-CoV and MERS-CoV, belongs to beta-coronaviruses and causes severe pneumonia in humans [1], and the same as SARS-CoV it invades human cells using an ACE-2 receptor and serine protease TMPRSS for S protein priming [2,3,4]. Genome sequencing of SARS-CoV-2 proved that it differs from two earlier discovered coronaviruses. It also revealed that it shares 96.2% of its genome with RaTG13 bat coronavirus [2]. This suggests that the novel coronavirus may have originated in bats and may have been transmitted to humans by an intermediate host (e.g., pangolin, turtle, snake).

It is known that n-CoV2 is spread by droplets and has been detected in stool [2,5]. Yang et al. reported general characteristics of patients with COVID-19 based on seven articles collecting a total of 1576 Chinese participants [5]. The major and most common infection symptoms were fever (91.3% of patients), cough (67.7%), fatigue (51%) and dyspnea (30.4%). Other symptoms such as headache, dizziness, abdominal pain, diarrhea, nausea, vomiting, taste disorders and oral mucosa lesions also were described, but less often [1,6,7]. These findings have also been confirmed by others [1,5,6,7]. The most common comorbidities were hypertension (21.1%), diabetes mellitus (9.7%), cardiovascular disease (8.4%) and respiratory system diseases (1.5%). It has been highlighted that significant heterogeneities in estimates of hypertension and cardiovascular disease (CVD) exist between articles and that severely affected groups of patients are at higher risk for CVD, hypertension and respiratory system diseases [5]. Cardiac injury alone, defined as elevated high-sensitivity cardiac troponin I (hs-cTnI) or new echocardiographic or ECG changes, were present in 7.2% of patients overall, and 22% of patients required intensive care unit (ICU) care in a group described by Wang et al. [6]. All authors agreed that age and comorbidities are both connected with the critical course of the illness and with higher risk of death in COVID-19 infection.

In recent articles, the presence of SARS-CoV2 RNA in the heart tissue was proven [8,9], yet there is still no evidence of its direct cardiotropic cell entry. On the other hand, several plausible articles describe cardiovascular system involvement during the course of the illness. Possible cardiovascular manifestations of COVID-19 include: (1) acute coronary syndrome (STEMI or NSTEMI) reported with obstructive, nonobstructive or no coronary artery disease (CAD); (2) acute myocardial injury without obstructive CAD; (3) arrythmias; (4) cardiac failure with or without cardiogenic shock; (5) pericardial effusion with or without tamponade; and (6) thromboembolic complications [10].

In June 2020, an article by Schiavone et al. [11], describing possible pathophysiological links between SARS-CoV-2 infection and acute coronary syndromes, evaluating the best strategy for ACS (acute coronary syndrome) management, was published. Some similar topics, such as highlighting the role of increased oxygen demand, the pro-inflammatory state during infection and the influence of other respiratory tract infections in ACS pathogenesis, were discussed. While Schiavone focuses on management of ACS during the pandemic, which is of high importance, this article presents a detailed description of the role of inflammation not only in ACS but also atherosclerosis, adding new information that was not included previously. Emphasis should be put on the fact that multiple articles are being published on a daily basis, making our knowledge ever more complete and we acknowledge that the data available at the time of this writing may be obsolete in the future. The aim of this paper is to present an insight into possible correlations between SARS-CoV-2 infection, atherosclerosis and acute coronary syndromes.

## 2. Methodology

**Figure 1:**
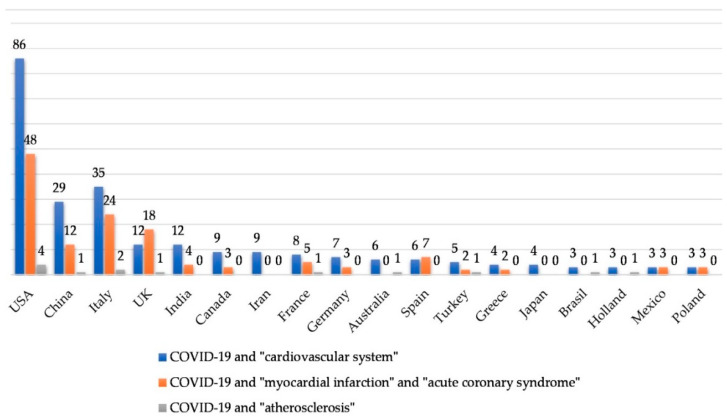
Analysed articles by country.

**Figure 2:**
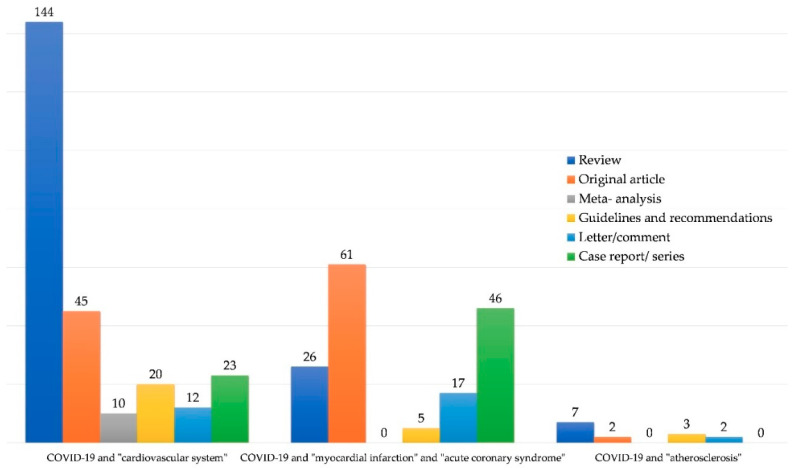
Types of articles analysed by search words.

We performed an analysis of the PubMed, Cochrane, Google Scholar and MEDLINE databases. We used the terms “COVID-19”, “SARS-CoV-2”, “n-CoV2” linked with “cardiovascular system”, “atherosclerosis”, “heart”, “AMI”, “ACS”, “acute myocardial infarction” and “acute coronary syndrome”. Most articles in similar fields (for example “cardiovascular system” and “heart” as well as “AMI” and “acute myocardial infarction”) overlapped and the decision was made to use the most representative key words, which were: “COVID-19”, “SARS-CoV-2”, “n-CoV2” linked with “cardiovascular system”, “atherosclerosis”, “acute myocardial infarction”, and “acute coronary syndrome”. As of September 15, 2020, the results were as follows: for “COVID-19” (and substitute words) and “cardiovascular system” we obtained 687 results; for “COVID-19” (and substitute words) and “myocardial infarction” together with “COVID-19” (and substitute words) and “acute coronary syndrome” we obtained 328 results; for “COVID-19” (and substitute words) and “atherosclerosis” we obtained 57 results. Some of them did not fulfill the search criteria or were concerned with the field of neurology. Only articles written in English, German and Polish were analyzed, for a total number of 432 papers. The distribution of articles by country is presented in Figure 1 (by keywords) and Figure 2 (by article type). Countries publishing fewer than two articles were not presented in the chart.

## 3. Inflammation and Atherosclerosis

Nowadays, atherosclerosis is defined as an inflammatory process occurring as a response to accumulation of lipids within the arterial wall. Some factors, like hypercholesterolemia and hypertension, increase wall permeability for low-density lipoproteins (LDLs) and leucocytes (monocytes and lymphocytes), which accumulate in the subendothelial space. Their oxidation, activated upregulation of adhesive molecules, such as vascular cell adhesion molecule-1 (VCAM-1), creation of foam cells and infiltration by CD4 T cells leads to a constant inflammatory response [12]. The result is plaque building and it later progresses to a vulnerable plaque, which is at high risk of thrombosis. Vulnerable plaques are divided into rupture-prone and erosion-prone plaques. The former are characterized by large plaque size, large necrotic core, neovascularization with intraplaque hemorrhage, increased adventitial inflammatory infiltration and a “spotty” pattern of calcifications [13,14,15]. Both can immediately lead to acute coronary syndromes.

## 4. Inflammation and Acute Coronary Syndromes

Inflammation plays an important role in triggering acute coronary syndromes (ACS) [16]. Patients with ACS present with more inflammatory markers in the blood, such as C-reactive protein, neutrophiles, procalcitonin, leucocytes, and have higher inflammatory activity within the entire coronary bed as well as more plaques with disrupted surfaces [17,18,19,20]. On the other hand, culprit lesions (those responsible for triggering ACS) are characterized by more advanced infiltration of inflammatory cells such as macrophages, T-lymphocytes and neutrophiles than any other coronary lesion [16,21]. They produce cytokines, proteases, factors affecting coagulation and vasoconstriction process and oxygen radicals. Additionally, a procoagulant effect of infection itself and hemodynamic changes cannot be avoided. All of these components may co-act, increase endothelial damage, leading to fibrous cap disruption and finally the formation of a coronary thrombus [21]. It should also be mentioned that increased CRP and IL-6 levels alone are independent risk factors for acute coronary syndromes [22,23]. Finally, an autopsy study by Madjid et al. revealed increased macrophage and dendritic cell density in atherosclerotic plaques as well as increased infiltration of macrophages and T-cell lymphocytes in adventitia and periadventitial fat in coronary arteries of patients with systemic infection compared to patients dying without infection [24]. This proves that systemic inflammation affects coronary tree plaques.

## 5. Acute Respiratory Tract Infections and Acute Coronary Syndromes

Inflammation and infection may promote atherosclerotic disease, as was first described in mice [25]. Later, a higher risk of acute myocardial infarction and stroke in humans with respiratory tract infection was confirmed [26]. In the current literature a 2- to 6-fold increase in cardiovascular events (acute myocardial infarction and stroke) was described after an acute respiratory tract infection. Studies trying to identify particular pathogens indicate influenza [27] and S. pneumoniae infections [28] as the most common causes of cardiovascular complications [29]. There are two mechanisms co-existing and exacerbating each other’s action: First, an inflammatory response of the organism in reaction to an acute respiratory infection which promotes atherosclerosis and plaque rupture; and second, the procoagulant and hemodynamic effect of infection itself resulting in possible thrombus and ischemia [30]. These potential mechanisms are presented in Table 1.

## 6. Inflammation and COVID-19

Unfortunately, in patients with COVID-19, inflammation becomes a bigger problem. Dysregulation affecting T-lymphocytes and an uncontrolled inflammatory process in patients with COVID-19 are the main concerns connected with immunopathology. It is known that regulatory lymphocytes are responsible for maintaining homeostasis, preventing exaggerated inflammation after infection, and providing an overall efficient immunological response [31]. Blood samples of patients infected with SARS-CoV-2 showed lowered blood levels in both T-lymphocyte, helper and regulatory lines [32], which may result in excessive and uncontrolled inflammation. Additionally, a cytokine storm seems to be implicated in two main causes of death: (1) acute respiratory distress syndrome (ARDS) and, much more uncommonly, (2) secondary hemophagocytic lymphohistiocytosis (sHLH).

A cytokine storm can be defined as an inordinate or uncontrolled production of cytokines. The term was used for the first time in the early 1990s, when muromonab-CD3 (OKT3) was introduced into the clinic as an immunosuppressive drug preventing transplant rejection [33,34]. A cytokine storm is a serious complication of immunotherapy and some autoimmune, neoplastic or infectious diseases, for example SARS [35], influenza [36] and SARS-CoV2 [37]. A group of 41 COVID patients, including 13 patients treated in intensive care units (ICU) and 28 non-ICU patients, was tested for inflammatory factors in the blood [1]. In this research, Huang et al. reported that blood levels of IL-1B, IL-1RA, IL-7, IL-8, IL-9, IL-10, fibroblast growth factor (FGF), granulocyte-colony stimulating factor (G-CSF), interferon-γ (IFNγ), interferon-γ-inducible protein (IP10), monocyte chemoattractant protein (MCP1), macrophage inflammatory protein 1 alpha (MIP1A), tumor necrosis factor (TNFα) and vascular endothelial growth factor (VEGF) were increased in both groups when compared to healthy individuals. Furthermore, ICU patients showed higher concentrations of G-CSF, IP10, MCP1, MIP1A and TNFα than non-ICU patients. A higher level of interleukin-6 alone was also proven to be associated with increased mortality [38]. These results suggest that cytokine storm is associated with a higher mortality rate and a more severe course of disease. Interestingly, serum levels of α hydroxybutyrate hydrogenase (αHBDH) which is associated among others with the size of damaged heart tissue [39], were higher in a relevant subset of patients with COVID-19 [40,41].

As mentioned above, a cytokine storm seems to be involved in two main causes of death: (1) acute respiratory distress syndrome (ARDS) and (2) secondary hemophagocytic lymphohistiocytosis (sHLH). ARDS is an acute lung failure caused by noncardiogenic pulmonary oedema. Its pathophysiology consists of an excessive inflammatory response and destabilization of VE-cadherin bonds resulting in increased endothelial permeability, which is caused by increased concentration of thrombin, tumor necrosis factor- α (TNF- α), vascular endothelial growth factor (VEGF) and intense leukocyte signaling [42,43]. The Berlin Definition of ARDS is presented in Table 2. [44].

HLH (hemophagocytic lymphohistiocytosis) is caused by a permanent stimulation of Toll-like receptors and antigen-presenting cells combined with an uncontrolled T-cell activation, which finally clinically manifests with multi-organ disfunction. It is often difficult to differentiate from sepsis [45,46] as secondary HLH in adults is mostly induced by viral infection [37]. Diagnostic criteria are presented in Table 3 [47].

## 7. COVID-19 and Cardiovascular Disease

Symptoms of COVID-19 are more severe among patients with cardiovascular disease. This is probably correlated with increased ACE-2 secretion when compared with healthy individuals [48]. As mentioned above, cardiac injury affected 7.2% of patients overall and 22% of ICU patients in a group of 138 patients; cerebrovascular disease was observed in 16.7% of the group [6]. Hypertension and CVD were present in 21.1% and 8.4% of patients, respectively, in a cohort of 1576 infected participants [5]. It was also reported that pre-existing heart disease, obesity and diabetes mellitus are clearly associated with an adverse effect of SARS-CoV-2 infection [5,8,9]. Furthermore, patients with cardiac injury have a higher mortality rate compared to those without cardiac injury (51.2% vs. 4.5%, respectively) [49]. The biggest study in China, using a report from the Chinese Center for Disease Control and Prevention, found an overall case-fatality rate (CFR) of 2.3%. Interestingly, but not surprisingly when looking above, the CFR was higher for patients with mentioned comorbidities: 10.5% for patients with cardiovascular disease, 7.3% for diabetic patients and 6.0% for patients with hypertension [50]. A multi-center study in Italy evaluated the connection of troponin levels with mortality rate in Italian patients, whose characteristics (older population with higher prevalence of comorbidities) made them predisposed to a higher risk of death and complications when compared to the Chinese population. In a total of 614 patients, 278 presented elevated troponin levels. The prevalence of comorbidities in this group was as follows: 65.9% had hypertension, 29.7% diabetes mellitus, 38.5% dyslipidemia, 22.8% heart failure, 24.3% atrial fibrillation, 31.5% coronary artery disease and 11.2% chronic obstructive pulmonary disease. Elevated troponin levels were associated with 71% increase of in-hospital death and 2-fold increase in major complications during hospitalization. This is the first study to evaluate a European population [51]. Consistent results were obtained by Lala et al. studying a group of nearly 3000 patients. Additionally, they reported that: (1) patients with earlier cardiovascular disease are more likely to have myocardial injury; (2) patients with increased age, body mass index and higher illness severity (assessed with CURB-65 score) are at higher risk of death; (3) statin use is associated with improved survival rate; and (4) myocardial injury is often observed among COVID-19 patients, but mostly presented with low-level troponin elevation [52]. Statistics show us clearly that the cardiovascular system plays an important role in COVID-2019.

However, instead of earlier CVD it should be noticed that the cardiovascular system is impacted in many patients with COVID-19. Clinics indicate myocardial injury as the main presentation which can be manifested by: elevated hs-cTnI level, arrhythmias, ST segment elevation and/or depression on ECG in the absence of obstructive coronary artery disease [8,53]. Other possible cardiovascular manifestations are: acute coronary syndrome, arrythmias, cardiac failure with or without cardiogenic shock, pericardial effusion with or without tamponade and thromboembolic complications [10]. An autopsy study on 23 patients reported in the United States describes pathologists’ findings in the cardiovascular system: viral uptake into interstitial, perivascular and endothelial cells; endothelitis; myocarditis or pericarditis in some cases; microvascular dysfunction; direct or indirect damage to cardiomyocytes [8]. Viral myocarditis should be treated and observed cautiously as it can present with symptoms that imitate acute coronary syndromes such as chest pain, clinical heart failure and electrocardiogram abnormalities. It is also dangerous since it can lead to a sudden worsening of cardiac contractibility in its fulminant form.

Arrhythmia is a common complication in COVID-19 patients and a common symptom of cardiac injury in ACS as well. It is also a common manifestation of viral infection which is experienced in everyday practice. Arrhythmogenicity in viral infections is said to be based on myocardial inflammation, which directly leads to ion channel dysfunction or electrophysiological and structural remodeling [54]. A description of a subset of patients primarily suffering from palpitations or a feeling of chest tightness suggested a possibility that their problem is of cardiac origin rather than SARS-CoV-2 infection [48]. Wang et al. reported that among the 138 patients, 23 (16.7%) of those had arrhythmic complications and out of the 38 ICU patients, 16 (44.4%) were transferred due to this particular complication [6]. Unfortunately, there are no further data available concerning the kind of arrhythmia or what further procedures were carried out. Arrhythmias described in the literature on COVID-19 include atrial fibrillation, sinus bradycardia, ventricular tachyarrhythmias and torsade de pointes (TdP). Their origin is not yet explained, but possible mechanisms suggest that they are initiated by viral myocarditis affecting the cardiac conducting system or that they can be an effect of medication (QT-prolonging medications causing ventricular arrhythmias or TdP), hypoxemia and pulmonary disease, activated protein kinase C or direct oxidized calcium-/calmodulin-dependent protein II activity [54]. A case describing and analyzing the course of ventricular tachycardia storm in a female patient with COVID-19, pneumonia and heart failure (ejection fraction 34%) after STEMI in 2014, and ICD implantation for primary prevention, was described by Mitacchione et al. This patient experienced multiple ICD shocks due to ventricular arrhythmia during hospitalization. No device interventions were described before the time of infection. Although electrical storm has many triggers which cannot always be properly identified, this is the first case connecting the incidence of ventricular tachycardia and SARS-CoV-2 infection [55].

Acute cardiac injury and coagulation biomarkers should be observed due to their connection with increased mortality and morbidity in COVID-19 patients. Creatine kinase-myocardial band (CK-MB), brain natriuretic peptide (BNP), D-dimer and prothrombin time (PT) are connected with acute myocardial injury, in-hospital death and ICU admission. Similarly, a reduced platelet count is also observed in acute myocardial injury and increases in-hospital deaths [56]. However, the level of hs-cTnI must be interpreted carefully because it may not reflect irreversible myocardial injury caused by ischemia or inflammation. For example, Zhou et al. observed that an increase in its level was constantly getting higher with the length of illness in patients who did not survive [38]. It has been proven that levels of acute phase inflammatory markers like D-dimer, C-reactive protein, lactate dehydrogenase and procalcitonin are elevated among patients with higher troponin levels [52]. Furthermore, the rise in hs-cTnI and other inflammation biomarkers such as IL-6, d-dimers, ferritin and lactate dehydrogenase suggests a possibility that this condition is a cytokine storm or hemophagocytic lymphohistiocytosis rather than an isolated cardiac injury [4]. Another report defined cardiac injury as isolated elevation of troponin serum level regardless of ECG and echocardiography, which was a result of incomplete data (only 22 of 82 patients with cardiac injury underwent electrocardiographic examination) [49]. An article collecting a total of 112 COVID-19 patients reported myocarditis in 14 cases (12.5%) based on troponin elevation and echocardiographic changes (i.e., segmental wall motion abnormalities, left ventricular ejection fraction (LVEF) < 50%, or presence of left ventricular wall thickening >10 mm and/or pericardial effusion) and ECG changes (ST elevation or ST/T segment changes). Authors highlighted that all 14 patients also had increased CK-MB and NT-proBNP levels and none of them had LVEF< 40% [56]. In conclusion, it is easy to overinterpret a high sensitivity cardiac troponin level in patients with COVID-19, since it is also associated with acute myocardial injury, ICU admission, death during hospitalization and the severity of inflammation [57].

When it comes to acute coronary syndromes, it should be mentioned that proposed protocols concerning the management of ST-elevation myocardial infarction patients differ between China, who propose a strong consideration of prior thrombolysis [58], and the American College of Cardiology Interventional Council and the Society for Cardiovascular Angiography and Interventions, who recommend a percutaneous coronary intervention (PCI) as a routine procedure for STEMI patients [59]. Furthermore, a decreased number of patients hospitalized because of STEMI has been reported as well as a prolonged time for time components in STEMI patients, especially in the first medical contact since symptom onset [60,61]. This can be caused by the fact that the symptoms of myocardial infarction may be underestimated in the context of the pandemic and additionally intensified by psychological factors.

Cardiac complications seem to be less common in SARS than in COVID-19 [10]. Due to findings concerning patients twelve years after SARS-CoV infection, potentially modified lipid and lipid metabolism should also be taken into consideration as a source of cardiovascular system damage with a late onset. When compared to healthy individuals, patients with infection (SARS-CoV) history had increased free fatty acids, lysophosphatidylcholines, lysophosphatidylethanolamines and phosphatidylglycerol serum levels [62]. On the other hand there were also research limitations: a small study group (consisting of 25 patients) and inability to state whether patients with glucose intolerance had taken medication. However, Zheng et al. highlights cardiovascular protection due to COVID-19 infection [48].

## 8. COVID-19 and Atherosclerosis

There are four potential ways in which cardiac muscle and cardiovascular injury may be explained in a novel coronavirus infection: First, a direct effect of the virus on cardiomyocytes; second, a hypoxia induced by respiratory tract infection; third, myocardial interstitial fibrosis; and fourth, acute infection and immune response of the organism including a cytokine storm which may alone trigger an acute myocardial infarction [63,64]. Electrolyte imbalance and the adverse effects of certain medications are other factors that may in addition force the heart muscle to work harder [64]. Nevertheless, possible disruption in coronary microcirculation with ischemic consequences remains putative, which was already suggested by Akhmerov et al. [63].

Angiotensin converting enzyme 2 (ACE2) is highly expressed in the lungs as well as in the heart; it is located in macrophages, endothelium, cardiac fibroblasts, smooth muscle cells and myocytes. Its activity is increased in patients after myocardial infarction (MI) [65], diabetes [66] and hypertension [67], all conditions predisposing patients to a fatal COVID-19 outcome, connected with the development of atherosclerosis and a higher risk of myocardial infarction. In theory, SARS-CoV-2 infects a similar spectrum of cells as SARS-CoV in laboratory conditions [3]. Based on this information it can be expected for both viruses to behave similarly in vivo. They can infect mainly pneumocytes and macrophages in lungs and extrapulmonary tissues with ACE-2 expression [4], such as, among others, the heart. In a recent article, a dipeptidyl peptidase-4 (DPP-4) receptor was suggested to facilitate cell entry for SARS-CoV-2 as it has similar spike glycoprotein to MERS-CoV, which invades human cells using this receptor [68]. A DPP-4 receptor is known for its role in promoting progression of atherosclerotic plaques by influencing migration of monocytes and downregulation of adiponectin and stromal-derived factor 1 (SDF-1) as well as inhibiting the GLP-1R signaling pathway in vein endothelial cells. It was also proposed that people with increased lipoprotein A levels are at higher risk for developing cardiovascular complications. This hypothesis needs to be tested in the future [69].

**Figure 3:**
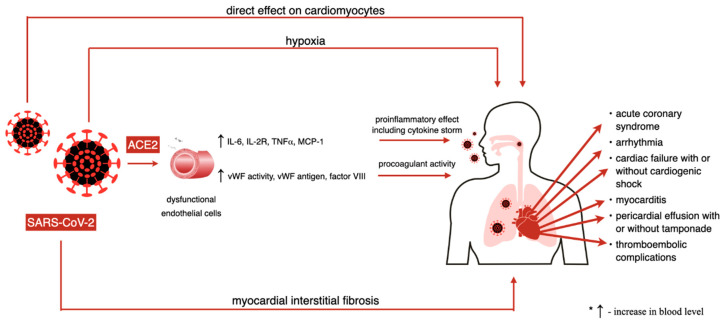
Effect of SARS-CoV-2 on cardiovascular system.

Inflammation caused by SARS-CoV-2 (described above) may affect atherosclerotic plaques, induce prothrombotic changes in blood and endothelium and lead to their instability, causing MI [21]. Endothelial activation is likely to be mediated by the ACE2 receptor. The first result regarding infection is a reversible dysfunction of endothelial cells and then their irreversible dysfunction characterized by apoptosis and necrosis. COVID-19-induced endothelitis is connected with an increase of circulating proinflammatory cytokines (IL-6, IL-2R, TNF- α) and chemokines (MCP-1) released by dysfunctional endothelial cells and an increase of von Willebrand factor antigen, von Willebrand factor activity and factor VIII levels in the blood. These factors may participate in leukocyte recruitment within the vasculature and directly lead to anticoagulant and procoagulant imbalance, resulting in a continuous proinflammatory and procoagulant state [70]. It is known that thromboembolic complications are common among COVID-19 patients. A presence of microthrombi was described in lungs, prostate and kidneys [70]. The pathophysiology and incidence of pulmonary embolism, one of the most common complications, is especially being studied during SARS-CoV-2 infection. Yet, there is still no evidence of the presence of thrombi in the coronary bed and an influence of COVID-19 infection on atherosclerotic plaque progression. This matter needs to be evaluated in the future. An effect of SARS-CoV-2 on cardiovascular system is presented in Figure 3.

Acute systemic inflammation can directly depress myocardial function and increase left ventricular afterload. Hypoxemia decreases myocardial oxygen delivery and raises pulmonary arterial pressure and right ventricular afterload [71]. An excessive and dysregulated immune response becomes an immunopathology also in the case of other human coronaviruses [72]. Lastly, lymphopenia, a condition observed in many COVID-19 patients [6], was proven to support the development of atherosclerosis leading to cardiovascular complications [73,74].

## 9. COVID-19 and Cardiovascular Drugs

Due to the fact that patients with underlying CVD have higher ACE2 expression, the suspicion of unfavorable influence of angiotensin-converting enzyme inhibitors (ACEI) and angiotensin receptor blockers (ARBs) was raised early in the pandemic. In normal conditions angiotensin I is converted to angiotensin II under the influence of ACE. Furthermore, angiotensin I under the influence of ACE2 converts to angiotensin 1-9 acting on AT_2_ R and angiotensin II to angiotensin 1-7 acting on Mas receptor (MasR). Both receptors have a beneficial effect on cardioprotection. ACE2/Ang1-7/MasR axis as well as ACE2/Ang1-9/AT_2_ R axis act as counter-regulators of the renin- angiotensin system. Angiotensin II itself shows a vasoconstrictive, proinflammatory, pro-oxidative effect and increases salt and water resorption resulting in cell proliferation, fibrosis and hypertrophy [75]. There have been two hypothesis: (1) ACEI/ARBs increase the risk for SARS-CoV-2 infection by the upregulation of ACE2 and (2) the therapeutic role of ARBs in COVID-19 in their tissue protective role (due to increased angiotensin 1-7 level) and inhibition of angiotensin II-induced inflammation and acute lung injury [76]. Some of the most recent research, carried out on large populations, showed that there was no negative influence on the course of SARS-CoV-2 infection by ACEI/ARBs, although a more frequent use of these drugs was observed among COVID-19 patients probably because of the higher prevalence of CVD in this group [77,78]. Because of the possible protective actions of ACE2 on lung injury, there are studies suggesting human recombinant soluble ACE2 (hrsACE2) may be a potential drug for COVID-19 [79,80]. Chinese medicine may play a role in COVID-19 treatment as well. Glycyrrhizin (applied in chronic hepatitis, extracted from Glycyrrhiza radix), hesperetin (a biflavonoid extracted from Citrus anrantium and pericarpium Ciri Reticulae) and baicalin (purified from Scutellaria bailacensis Georgi with antioxidative, anti-inflammatory and anti-apoptosis effect) are expected to bind or inhibit ACE2 [80].

Another important group in cardiac treatment are statins. They play an important role in lowering cardiovascular risk factors. Their pleiotropic effect, observed in various diseases, is based on the reduction of inflammatory response [81]. Lala et al. stated that statin use is associated with improved survival rate [52]. They may act as a SARS-CoV-2 main protease inhibitor [82] and their potential use in COVID-19 patients is being discussed. There are also current studies evaluating the anti-inflammatory effect of colchicine in COVID-19 [83,84], nitric oxide [85] and heparin [80,86]. These drugs may give us a double action in fighting against cardiovascular disease and COVID-19.

Last but not least is the role of diet in cardiovascular disease prevention. Nutrition is proven to improve patient outcomes. Numerous diets have been proposed to patients with an anti-inflammatory and immunomodulatory effect on the organism. One of them is the Mediterranean diet, focusing on consumption of un-processed or minimally processed food. Furthermore, vitamins and trace elements like vitamin C, vitamin D and zinc are proposed to be beneficial in SARS-CoV-2 infection [80,87].

## 10. Discussion

When atherosclerosis became an inflammatory disease, several infection agents were described to intensify its progression. In 1909 Osler wrote, “It is exceptional to find no patches of arterial degeneration in any body postmortem, and even children may show some slight foci of fatty degeneration”. This disease has been observed for ages, yet we do not know everything about it. Similarly, in the COVID-19 pandemic, new discoveries remain to be made. As said above, multiple articles are being published on a daily basis, making our knowledge ever more complete and we acknowledge that the data available at the time of this writing may be obsolete in the future. Some dependencies between analyzed disease units are very complex which makes their discovery really difficult, especially when these correlations are not obvious and a large set of data needs to be analyzed. The methods of big data analysis, particularly deep learning and neuron networks, may be a solution of the problem in the future [88]. A study by Ambale-Venkatesh et al. shows that in silico analysis is possible in stratifying the cardiovascular risk in an ethnically different cohort [89]. There is also research trying to connect imaging with an in silico interpretation of obtained results in classification of atherosclerotic lesions [90] or prediction of coronary vessel occlusion [91]. Artificial intelligence has found a place in the COVID-19 pandemic as well, in diagnosis, detection and prevention of the infection, monitoring and development of the treatment, tracing the contacts of people, assessing case numbers and mortality and reducing the workload in the healthcare system [92]. On the other hand, these solutions are still new and need further research, improvements and evaluations. Some problems can only be solved with artificial intelligence, assuming that it would be optimal to use petabytes of global human resource data. It is a change in the way people think and practice science. A quote from Mark Twain, one of the most famous writers, considered the father of American literature, best describes this: “The only person who wants a change is a child with a wet diaper”.

## 11. Conclusions

While the link between inflammatory response, COVID 19 and atherosclerosis still remains unclear, there is evidence that suggests a more likely correlation between them. Practitioners’ efforts should be focused on the prevention of excessive inflammatory response and possible complications, while there are limited specific therapeutic options against SARS-CoV-2. Furthermore, special attention should be paid to cardioprotection during the pandemic.

## Figures and Tables

**Table 1:** Potential mechanisms that may trigger acute coronary syndrome during respiratory tract infection. Adapted from Bazaz et al. [30].

Process	Effect
Systemic host response to infection	Systemic inflammation and intense cytokine release
Local host response to infection	Local vascular inflammation- inflammatory cells infiltrating atherosclerotic plaques;
	Plaque vulnerability- macrophage polarization, differentiation of T-helper lymphocytes, macrophage apoptosis
Thrombosis	Activation of coagulation system and platelets;
	Endothelial dysfunction
Haemodynamic effects	Peripheral vasodilatation especially in sepsis;
	Coronary vasoconstriction due to catecholamine release;
	Altered (increased) myocardial metabolic demand and hypoxaemia which may result in increased myocardial region vulnerable to ischaemia

**Table 2:** Berlin Definition of ARDS. Adapted from Ranieri et al. [44].

Criteria	Explanation		
Timing	Within 1 week of a known clinical insult or new or worsening respiratory symptoms		
Chest imaging ^a^	Bilateral opacities—not fully explained by effusions, lobar/ lung collapse, or nodules		
Origin of oedema	Respiratory failure not fully explained by cardiac failure of fluid overload Need objective assessment (e.g., echocardiography) to exclude hydrostatic oedema if no risk factor present		
Oxygenation ^b^	Mild	Moderate	Severe
	200 mmHg < PaO_2_/FiO_2_ ≤ 300 mmHg with PEEP or CPAP ≥ 5 cm H_2_O ^c^	100 mmHg < PaO_2_/FiO_2_ ≤ 200 mmHg with PEEP ≥ 5 cm H_2_O	PaO_2_/FiO_2_ ≤ 100 mmHg with PEEP ≥ 5 cm H_2_O

^a^ Chest radiography or computed tomography scan; ^b^ If altitude is higher than 1.000 m, the correction factor should be calculated as follows: [PaO_2_/FiO_2_ × barometric pressure/760]; ^c^ This may be delivered noninvasively in the mild acute respiratory distress syndrome group.

**Table 3:** Diagnostic guidelines for HLH. Adapted from Hayden et al. [47].

HLH- 2004 Criteria. Either 1 or 2 are Fulfilled:
(1) A molecular diagnosis consistent with HLH
(2) Diagnostic criteria for HLH fulfilled (5 out of the 8 criteria below):
(A) Initial diagnostic criteria: - Fever - Splenomegaly - Haemophagocytosis in bone marrow or spleen or lymph nodes - Hypertriglyceridemia and/or hypofibrinogenemia: Fasting triglycerides ≥3.0 mmol/L (i.e., ≥265 mg/dL); Fibrinogen ≤1.5 g/L - Cytopenias (affecting ≥2 of 3 lineages in the peripheral blood): Haemoglobin <90 g/L (in infants <4 weeks <100 g/L); Platelets <100 × 10^9^/L; Neutrophils <1.0 × 10^9^/L
(A) New diagnostic criteria: - Low or absent NK- cell activity - Ferritin ≥500 mg/L - Soluble CD25 (i.e., soluble IL-2 receptor) ≥2400 U/mL
